# Synovial fluid monocyte-to-lymphocyte ratio in knee osteoarthritis patients predicts patient response to conservative treatment: a retrospective cohort study

**DOI:** 10.1186/s12891-024-07475-1

**Published:** 2024-05-14

**Authors:** Jong Min Lee, Sumin Lim, Gunoo Kang, Jun Young Chung, Hee-Woong Yun, Yong Jun Jin, Do Young Park, Jae-Young Park

**Affiliations:** 1 Department of Orthopaedic Surgery, School of Medicine, Ajou University, Suwon, South Korea; 2 Cell Therapy Center, Ajou Medical Center, Suwon, Republic of Korea; 3 Leading Convergence of Healthcare and Medicine, Ajou University, Institute of Science & Technology (ALCHeMIST), Suwon, Republic of Korea; 4 Department of Orthopaedic Surgery, Uijeongbu Eulji Medical Center, Eulji University School of Medicine, Uijeongbu-si, Republic of Korea; 5 Department of Orthopaedic Surgery, CHA University, CHA Bundang Medical Center, Seongnam-si, Gyeonggi-do Republic of Korea

**Keywords:** Osteoarthritis, Monocyte, Monocyte to lymphocyte ratio, Biomarker

## Abstract

**Background:**

Biomarkers that predict the treatment response in patients with knee osteoarthritis are scarce. This study aimed to investigate the potential role of synovial fluid cell counts and their ratios as biomarkers of primary knee osteoarthritis.

**Methods:**

This retrospective study investigated 96 consecutive knee osteoarthritis patients with knee effusion who underwent joint fluid aspiration analysis and received concomitant intra-articular corticosteroid injections and blood tests. The monocyte-to-lymphocyte ratio (MLR) and neutrophil-to-lymphocyte ratio (NLR) were calculated. After 6 months of treatment, patients were divided into two groups: the responder group showing symptom resolution, defined by a visual analog scale (VAS) score of ≤ 3, without additional treatment, and the non-responder group showing residual symptoms, defined by a VAS score of > 3 and requiring further intervention, such as additional medication, repeated injections, or surgical treatment. Unpaired t-tests and univariate and multivariate logistic regression analyses were conducted between the two groups to predict treatment response after conservative treatment. The predictive value was calculated using the area under the receiver operating characteristic curve, and the optimal cutoff value was determined.

**Results:**

Synovial fluid MLR was significantly higher in the non-responder group compared to the responder group (1.86 ± 1.64 vs. 1.11 ± 1.37, respectively; *p* = 0.02). After accounting for confounding variables, odds ratio of non-responder due to increased MLR were 1.63 (95% confidence interval: 1.11–2.39). The optimal MLR cutoff value for predicting patient response to conservative treatment was 0.941.

**Conclusions:**

MLR may be a potential biomarker for predicting the response to conservative treatment in patients with primary knee osteoarthritis.

## Background

Osteoarthritis (OA) is a major health concern worldwide [1, 2]. Approximately 32.5 million people in the United States are estimated to have OA, and the medical costs attributable to OA are up to $373.2 billion and increasing every year [3–5]. For knee osteoarthritis (KOA), global prevalence is 22.9% in individuals aged ≥ 40 years [6].

While our knowledge regarding the pathophysiology, risk factors, and evidence-based interventions for OA continues to increase, monitoring disease progression and treatment response remains a challenge that ultimately affects clinical decision-making [7]. Well-established OA treatment guidelines such as OARSI and ESCEO guidelines recommend treatments in decreasing order of evidence in a “trial and error” manner; AAOS guideline (3rd edition) and 2019 ACR guideline merely enlist treatments with strong and conditional recommendations [8–11]. Unfortunately, treatment decision making in OA still largely relies on the experience and preferences of clinicians.

Therefore, the development of predictive biomarkers, is in great demand. Potential OA-specific biomarkers such as cartilage oligomeric matrix protein (COMP), tumor necrosis factor-α (TNF-α), nucleotide-binding oligomerization domain-like receptor containing protein 3 (NLRP3), cross-linked C-telopeptide (CTX), and microRNAs are being investigated, with some utilized in clinical trials [12–15]. However, such biomarkers have yet to show sufficient clinical significance in predicting treatment response and are often difficult to test in everyday clinical settings [7, 14].

Considering the pathophysiology of OA and ease of obtaining synovial fluid during KOA treatment in patients with joint effusion, synovial fluid analysis could be a promising source of novel predictive biomarkers. OA is often present in a chronic low-grade inflammatory state with innate immunity, which plays a major role in its pathophysiology and progression [12, 16–20]. Macrophages act as key modulators of OA-associated inflammation, and their populations are subdivided into pro-inflammatory and immunomodulatory tissue-resident macrophages [16, 21, 22]. A shift towards the pro-inflammatory macrophages, especially Ly6C^high^ monocyte-derived macrophage, produces inflammatory cytokines (alarmins, IL-1, and TNF-α), stimulates production of matrix metalloproteinases, and induces cytokine profile change in synovial fluid with high levels of IL-1β, IL-6, and IL-8. Stimulated chondrocytes produce more ECM-degrading enzymes and trigger a vicious inflammatory cycle between adipose tissue, chondrocytes, neutrophils, and activated macrophages, thus precipitating into subchondral bone remodeling, osteophyte formation, and other hallmarks of osteoarthritis [23].

Synovial fluid analysis provides cell counts of monocytes, neutrophils, and lymphocytes in the affected joint. Recent studies have shown that certain cell ratios, such as the neutrophil-to-lymphocyte ratio (NLR) or monocyte-to-lymphocyte ratio (MLR), predict treatment response in different diseases, including various cancers and rheumatoid arthritis (RA) [24–32]. These ratios reflect the inflammatory microenvironment in which local cells, including tumor cells, are present, by which apoptosis of these cells is inhibited and angiogenesis is promoted. RA, which shares the same pathophysiologic process as Ly6C^high^ monocyte-derived macrophage invasion, is more difficult to treat in patients whose peripheral blood NLR levels are higher [24]. However, the clinical value of these cell ratios is not well known in primary KOA [33–35]. Therefore, we aimed to investigate whether peripheral blood and synovial fluid cell counts and their ratios (NLR and MLR) could act as predictive biomarkers for treatment response in primary KOA.

We hypothesized that a higher NLR and MLR in the peripheral blood and synovial fluid would correlate with a worse treatment response in patients with KOA.

## Methods

This study was approved by the Ethics Committee of Ajou University Hospital. (No. AJOUIRB-DB-2023-025)

### Patient selection and study design

Consecutive patients who underwent joint aspiration for knee effusion between January 2018 and March 2022 were retrospectively investigated in the outpatient clinic of a single center. The inclusion criteria for the present study were as follows: (1) patients > 40 years; (2) Kellgren-Lawrence (KL) grade II or III; and (3) a follow-up period of > 6 months. Exclusion criteria for the present study were as follows: (1) synovial RBC count of > 1500 (×10^9^/L); (2) aspiration amount none or too small to attain cell counts or a specimen with cellular degeneration; (3) patients diagnosed with septic arthritis, inflammatory arthritis such as rheumatoid arthritis and gouty arthritis, or post-traumatic osteoarthritis; (4) valgus alignment and isolated lateral compartment knee OA; (5) history of recent corticosteroid injection within 6 months; (6) history of concurrent fracture; (7) knee surgery before aspiration; and (8) patients who could not receive oral anti-inflammatory medication due to various medical conditions.

Patients diagnosed with primary osteoarthritis accompanied by knee effusion underwent joint aspiration of the involved knee in the outpatient clinic. Joint fluid analysis and blood tests were performed for the differential diagnosis. As a standard of care, patients were given a concomitant intra-articular corticosteroid injection (triamcinolone acetonide 40 mg alone), and non-steroidal anti-inflammatory drugs (NSAIDs) (Celecoxib 200 mg QD) were prescribed for 1 month with a subsequent visit 1 month later, where additional treatment and follow-up plans were decided [9, 36]. All patients were previously given instructions for exercise and education on weight loss and lifestyle modifications as initial treatment attempts, following the aforementioned treatment guidelines. No physiotherapy sessions or other ancillary injections (e.g., hyaluronic acid and platelet-rich plasma) were administered during the follow-up period.

The patients were divided into two groups. The first group was called the responder group. It was defined as the resolution of joint effusion and documented symptomatic improvement of a visual analog scale (VAS) score of ≤ 3 at 6 months of follow-up. The second group was called the non-responder group. It was defined as having residual symptoms up to 6 months of follow-up, defined as a VAS score of > 3 and receiving any of the following: [1] additional injections during the follow-up period; [2] moving up one step in the WHO analgesic ladder [37] due to unresolved pain; and [3] receiving any type of surgical treatment on the affected knee, such as total knee arthroplasty, unicompartmental knee arthroplasty, or tibial osteotomy.

Demographic variables, including age, sex, body mass index (BMI), and KL grade were collected for all patients. The KL grade was determined by an experienced orthopedic surgeon. The patient demographics are summarized in Table 1.

**Table 1:** Baseline demographics and potential markers of treatment response

Parameter	Responder (*n* = 41)	Non-responder (*n* = 55)	*p*-value
**Demographics**			
Age (Years)	62.5 ± 7.7	64.4 ± 9.0	0.273
Female	29 (70.7)	42 (76.4)	0.534
BMI	25.1 ± 3.7	25.9 ± 3.7	0.310
KL Grade			0.063
Grade 2	25 (61.0)	23 (41.9)	
Grade 3	16 (39.0)	32 (58.1)	
**Response Markers**			
WBC (SF)	179.8 ± 148.6	166.6 ± 153.5	0.674
Neutrophils (SF)	13.4 ± 30.3	7.5 ± 15.4	0.255
Monocytes (SF)	63.7 ± 74.5	86.3 ± 93.2	0.206
Lymphocytes (SF)	98.0 ± 90.7	67.0 ± 67.3	0.057
NLR (SF)	0.13 ± 0.36	0.16 ± 0.33	0.643
MLR (SF)	1.11 ± 1.37	1.86 ± 1.64	**0.020**
WBC (PB)	6.21 ± 1.60	6.42 ± 1.19	0.499
Neutrophils (PB)	3.51 ± 1.30	3.58 ± 0.98	0.757
Monocytes (PB)	2.12 ± 2.07	1.96 ± 2.08	0.706
Lymphocytes (PB)	2.01 ± 0.64	2.14 ± 0.58	0.292
NLR (PB)	1.90 ± 0.82	1.80 ± 0.66	0.520
MLR (PB)	1.16 ± 1.23	1.03 ± 1.04	0.569
ESR (PB)	11.2 ± 11.8	12.0 ± 9.82	0.711
hsCRP (PB)	0.45 ± 1.53	0.23 ± 0.94	0.395
MLR (SF/PB)	3.49 ± 10.1	9.31 ± 33.2	0.281
WBC x MLR (SF)	177.8 ± 265.6	324.8 ± 524.3	0.104

### Response marker selection

For all patients, the following laboratory markers were collected at baseline: synovial fluid cell count with differential white blood cell (WBC) count including neutrophils, macrophages, monocytes, lymphocytes, basophils, eosinophils, and mesothelial cells (×10^9^/L) and peripheral blood cell count with the same differential WBC count. Routine serum chemistry included erythrocyte sedimentation rate (ESR) (mm/h) and high-sensitivity C-reactive protein (hsCRP) (mg/dL).

Peripheral blood cell and WBC differential counts were analyzed by automated electrical impedance cell counter/hematology analyzer (Beckman Coulter Hematology analyzer/Siemens ADVIA); with an abnormal result, a clinical pathologist re-checked the blood count with microscopy. Synovial fluid cell and WBC differential counts were calculated by a clinical pathologist using a microscope and Neubauer counting chamber (Marienfeld Superior, 0.0025 mm^2^, 0.100 mm depth). The synovial smear was stained with modified Wright’s stain to accurately calculate the differential count. Samples with a WBC count of > 300 were reanalyzed by clinical pathologists under a microscope for differential WBC counts in percentages.

Synovial fluid and peripheral blood NLR were defined as the neutrophil count divided by the lymphocyte count. Synovial fluid and peripheral blood MLR were defined as monocyte counts divided by lymphocyte counts. All cell counts and ratios were investigated to determine statistically significant differences between the two groups.

### Statistical analysis

Descriptive statistics were calculated for the baseline characteristics of the study population. The unpaired t-test was used for mean comparison of age, BMI, and response markers, and the chi-square test was used for sex and KL grade. Univariable and multivariable logistic regression analyses were performed for baseline characteristics and potential response markers with 95% confidence intervals (CI) and adjusted odds ratios (ORs) were determined. A receiver operating characteristic (ROC) curve was constructed and the optimal cutoff value for response prediction was calculated using Youden’s index. All the statistical analyses were performed using SPSS version 21 (IBM, Chicago, Armonk, NY, USA).

## Results

**Fig. 1:**
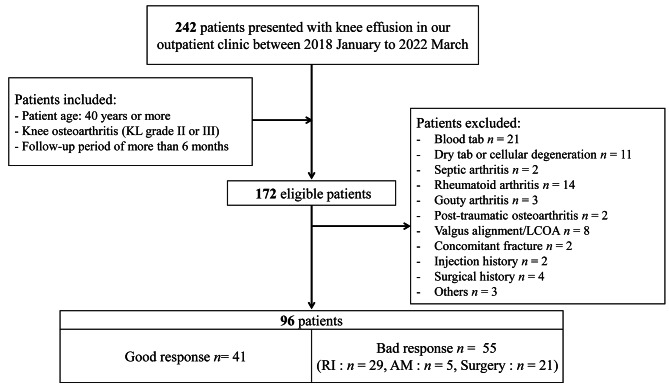
Flowchart of the study population. Abbreviations: KL, Kellgren-Lawrence grade; RI, repeat injection; AM, additional medication

A total of 254 consecutive patients with knee effusion who underwent joint aspiration at our outpatient clinic were enrolled in this study. A total of 187 patients met the inclusion criteria; of which, 91 patients were excluded, and 96 were analyzed. (Fig. 1)

**Fig. 2:**
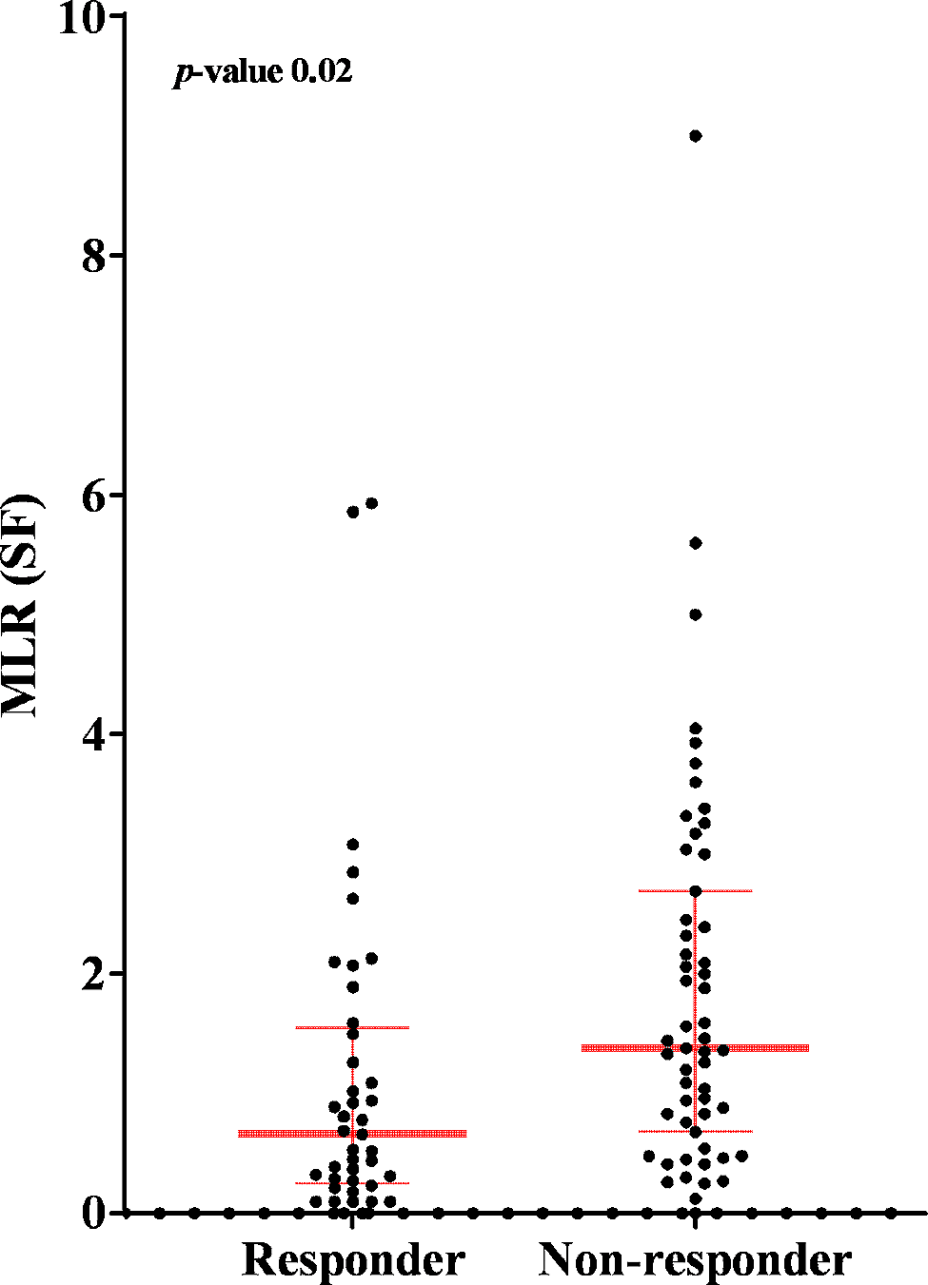
Scatterplot of synovial fluid MLR with median and interquartile range for “responder” and “non-responder” group. Abbreviations: MLR (SF), synovial fluid monocyte-to-lymphocyte ratio. *p*-value for unpaired T-test between the two groups

A total of 71 (74.0%) patients were female, and the mean age was 63.6 ± 8.5 years. The mean BMI was 25.6 ± 3.7. Fifty-five (57.3%) patients required additional treatment during their 6-month follow-up. The KL grade distribution among the two groups was not statistically different (*p*-value 0.063), albeit the non-responder group had more KL III patients compared to the responder group. (58.1% vs. 39.0%). None of the patients had leukocytosis or monocytosis in their peripheral blood. Among the basic demographic data and potential response markers investigated, synovial fluid MLR was significantly different between the two groups. The non-responder group showed higher synovial fluid MLR compared to that in the responder group (1.86 ± 1.64 vs. 1.11 ± 1.37, respectively; *p* = 0.02). (Fig. 2). The markers of response showed that the mean values for WBC, neutrophil, and monocyte counts and NLR in the synovial fluid were higher in the non-responder group than in the responder group; however, the differences were not statistically significant. The mean MLR value in the peripheral blood was lower in the non-responder group, but the difference was not statistically significant. The mean ESR and hsCRP levels were not significantly different between the two groups. (Table 1)

Univariable logistic regression and multivariable regression analyses for each demographic variable and synovial fluid MLR were performed (Table 2). The results showed that age was not a significant predictor of treatment response (OR 1.03, 95% CI 0.97–1.09, *p* = 0.333). Similarly, sex and BMI were not significant predictors of treatment response (OR 1.92, 95% CI 0.68–5.52, *p* = 0.226 and OR 1.12, 95% CI 0.99–1.27, *p* = 0.085, respectively). However, KL grade showed a trend towards significance, with KL grade 3 being associated with a higher odds of treatment non-response compared to KL grade 2 (OR 2.32, 95% CI 0.94–5.69, *p* = 0.067). The most significant predictor of treatment response was the MLR value in the synovial fluid, with higher MLR values being associated with higher odds of treatment non-response (OR 1.63, 95% CI 1.11–2.39, *p* = 0.013).

**Table 2:** Multivariable logistic regression analysis between the two groups to predict treatment response after conservative treatment

	Crude OR (95% CI)	*p*-value	Multivariable adjusted OR* (95% CI)	*p*-value
Age	1.03 (0.98, 1.08)	0.271	1.03 (0.97, 1.09)	0.333
Sex	1.34 (0.56, 3.34)	0.535	1.92 (0.68, 5.52)	0.226
BMI	1.06 (0.95, 1.19)	0.308	1.12 (0.99, 1.27)	0.085
KL Grade				
KL 2	1 (Ref)		1 (Ref)	
KL 3	2.17 (0.95, 4.96)	0.065	2.32 (0.94, 5.69)	0.067
MLR (SF)	1.47 (1.05, 2.07)	**0.026**	**1.63** (1.11, 2.39)	**0.013**

* Multivariable logistic regression analysis including patient age, sex, BMI, KL Grade, MLR (SF), and male sex as reference parameters

BMI, body mass index; KL Grade, Kellgren-Lawrence grade; MLR, monocyte-to-lymphocyte ratio; SF, synovial fluid

**Fig. 3:**
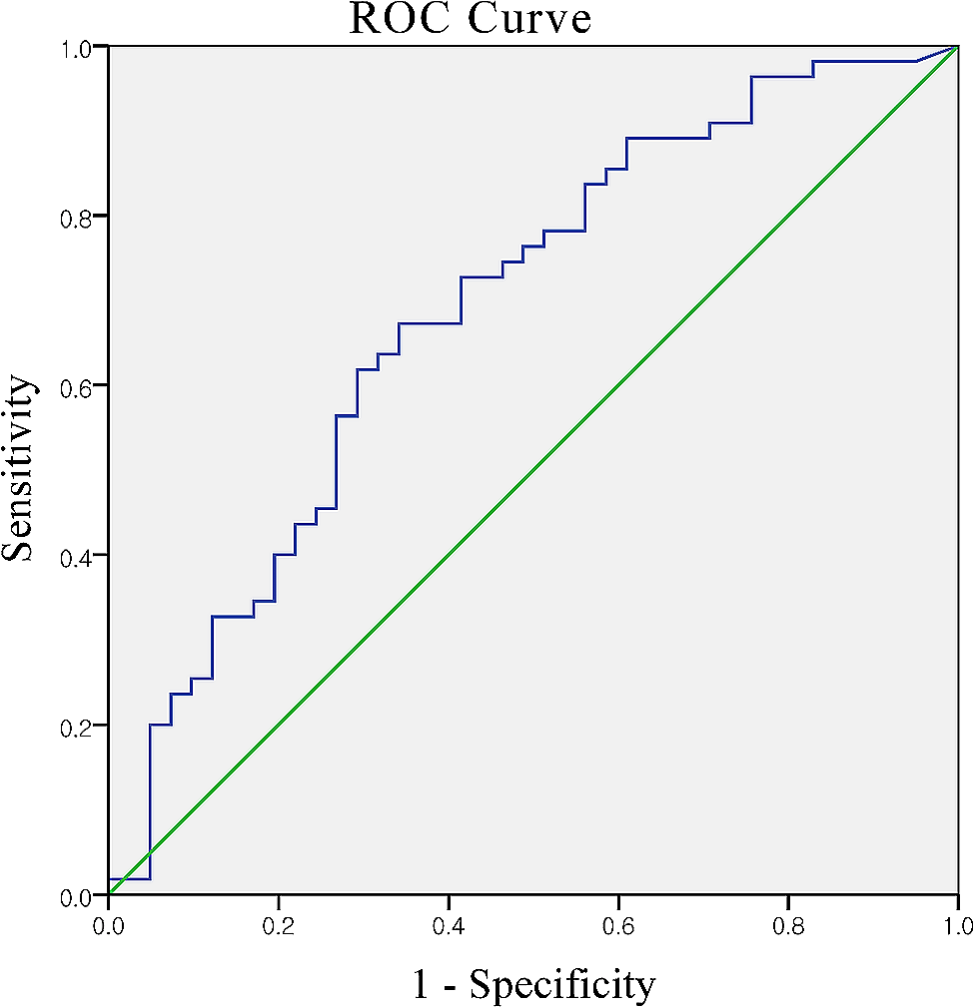
Receiver operating characteristic curve demonstrating sensitivity and specificity of synovial fluid monocyte-to-lymphocyte ratio

The cutoff value for synovial fluid MLR to predict treatment response according to the ROC curve was 0.941. With a sensitivity of 67.3% and specificity of 65.9%, the positive and negative predictive values were 72% and 58.7%, respectively, in our patient group. The area under the ROC curve was 0.69 (95% CI 58.0–79.7%). (Fig. 3)

## Discussion

This study aimed to investigate the role of synovial fluid analysis in identifying predictive biomarkers of primary KOA. We demonstrated that synovial fluid MLR may be higher in patients with a worse treatment response than in those with a better response to conservative treatment. This is the first study to validate the predictive value of the synovial fluid WBC ratio for conservative treatment in KOA patients, with a cutoff value of 0.941, indicating that synovial fluid MLR can be a quick and intuitive tool that clinicians can use in everyday outpatient clinics.

Circulating monocytes are pro-inflammatory cells that infiltrate the joints and initiate and propagate joint degeneration in both the acute and chronic stages of osteoarthritis. Synovium in OA induces activation, migration, and functional commitment of circulating classical CD14 + CD16- monocytes [38]. Numerous in vitro studies have found that monocytes/macrophages are the most abundant leukocytes in the OA synovium [21, 22]. Trajerova et al. found that KOA patients with high monocyte-macrophage immune phenotypes correlated with a worse clinical trajectory [39]. Gao et al. reported that patients with KOA showed higher blood MLR than healthy control participants [33]. Tasoglu et al. stated that higher peripheral blood NLR and lower NMR (neutrophil-monocyte ratio) with monocytes as the denominator were correlated with knee OA severity [35]. Higher synovial fluid MLR seen among non-responders in our study, therefore, may reflect a pro-inflammatory trait in KOA patients that is not easily amenable to anti-inflammatory measures taken; higher synovial fluid MLR should not be misconceived for more severe inflammation; however, this was not included in the scope of this study.

Previously reported OA biomarkers regarding prognosis in BIPEDS classification included synthesis and degradation related molecules such as serum COMP, urine CTX-II, and serum hyaluronan, as well as inflammatory markers such as hsCRP, IL-1β, IL-6, and prostaglandin E2 [40]. Continued research on urine CTX-II has reported an elevated risk of radiographic progression in patients with elevated baseline urine CTX-II levels [41]. Few studies have been conducted to predict the efficacy of the most common standardized anti-inflammatory interventions, such as intra-articular corticosteroid injection and NSAIDs [42]. The limited representation of serum and urine biomarkers in specific joints together with the heterogeneity of OA may have impeded such research. Synovial fluid obtained directly from the affected joint may provide a more specific clinical picture of the affected joint. We utilized cell count ratios from the synovial fluid of the affected joint, which are more readily available than the aforementioned biomarkers and are easily obtainable during joint aspiration or intra-articular injections. The clinical implication of our study is that with synovial fluid MLR, a practicable predictive biomarker, we may be able to anticipate treatment efficacy and accelerate decisions for treatment conversion, such as surgery.

The present study had some limitations. First, it was a retrospective study with a short-term follow-up period; however, we believe that its design was better fitted in the search for an applicable biomarker for the efficacy of treatment. Second, our allocation of patients to the treatment groups may have been arbitrary, with a binary division of patients based on the pain score (VAS) requiring additional intervention. However, because the primary goal of conservative care in patients with OA is pain reduction, the VAS was determined as the scale for patient designation. The difference in the KL grade between the two groups, although statistically insignificant, is also a potential limitation of the power of the current study. Application of our results may also be confined to varus knee OA, as we have excluded valgus knee OA patients due to differences in etiology, biomechanics, and treatment response. Finally, our biomarker, synovial fluid MLR, can only be gathered from patients with an attainable amount of effusion, which limits the accessibility of synovial fluid MLR to patients with KOA without joint effusion. However, it has the distinct advantage of synovial fluid biomarkers, which are more joint-specific and better reflect local pathophysiology. It is also effective when an intra-articular corticosteroid injection is administered.

## Conclusions

Synovial fluid MLR may be a potential biomarker for predicting patient responses to conservative treatment for primary KOA.

## Data Availability

The datasets used and/or analyzed in the current study are available from the corresponding author upon reasonable request.

## Abbreviations

MLR: Monocyte-lymphocyte ratio
NLR: Neutrophil-lymphocyte ratio
VAS: Visual acuity score
OA: Osteoarthritis
KOA: Knee osteoarthritis
COMP: Cartilage oligomeric matrix protein
TNF: Tumor necrosis factor
NLRP3: Nucleotide-binding oligomerization domain-like receptor containing protein 3
CTX: Cross-linked C-telopeptide
ECM: Extracellular matrix
KL: Kellgren-Lawrence grade
NSAIDs: Non-steroidal anti-inflammatory drugs
BMI: Body mass index
WBC: White blood cell
ESR: Erythrocyte sedimentation rate
CRP: C-reactive protein
CI: Confidence interval
OR: Odds ratio
ROC curve: Receiver operating characteristic curve
